# Supplementary figures and images for: dagLogo: An R/Bioconductor package for identifying and visualizing differential amino acid group usage in proteomics data
Source: PLoS One. 2020 Nov 6;15(11):e0242030. doi: 10.1371/journal.pone.0242030 (PMC7647101; doi:10.1371/journal.pone.0242030)

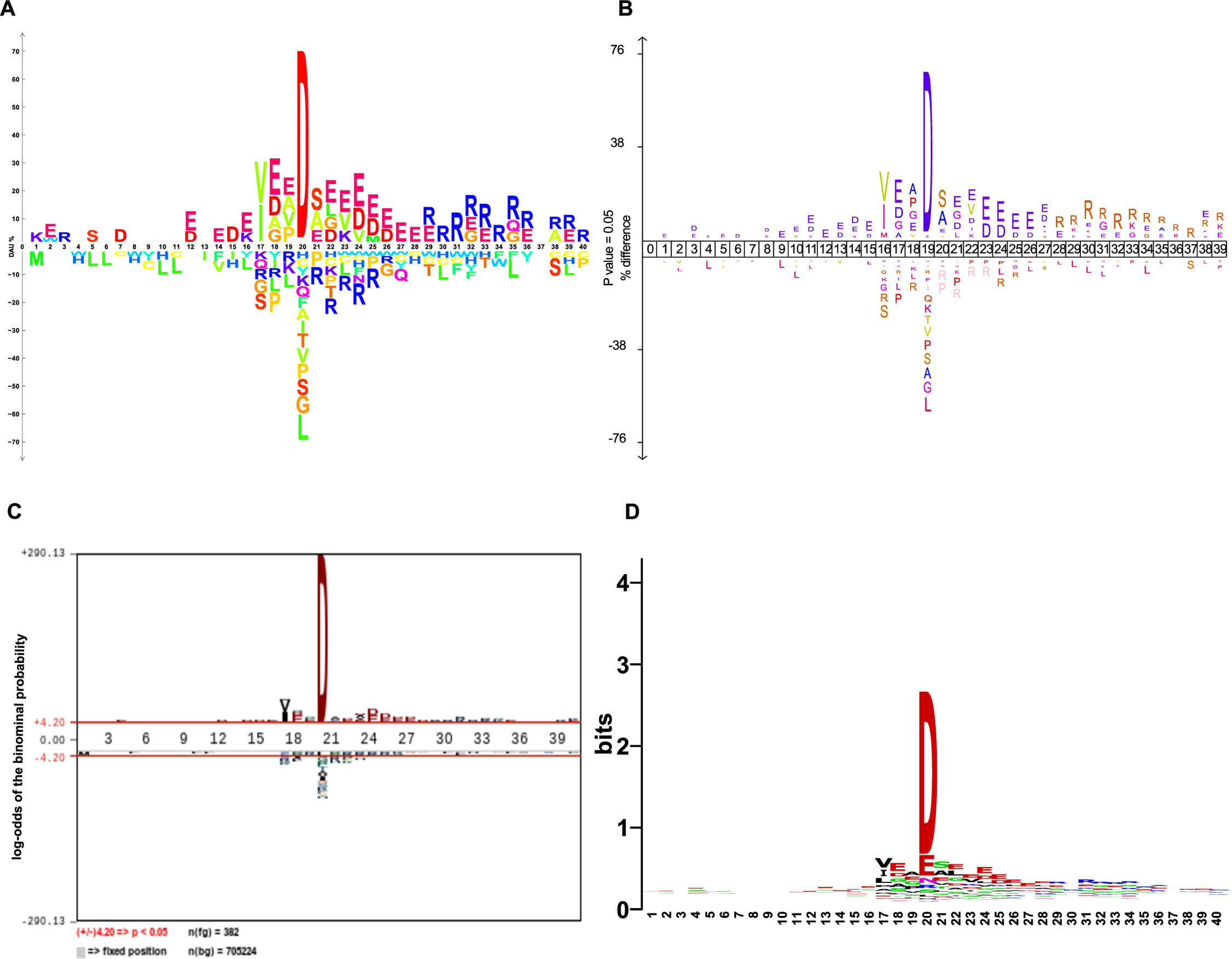

Supplement: S1 Fig — The same set of 416 aligned, equal-length subsequences of 30 residues centered on the cleavage sites was used as the input set. The same set of randomly sampled subsequences of 30 AA residues from the human reference proteome was used as the background for iceLogo (version 0.2), pLogo (version 1.1.0), and dagLogo (version 1.26.2). A uniform global background of equal probability (0.05) of each of the 20 natural AAs was used for WebLogo (version 2.8.2). (A) dagLogo, (B) iceLogo, (C) pLogo, and (D) WebLogo. (TIF) [file pone.0242030.s001.tif]

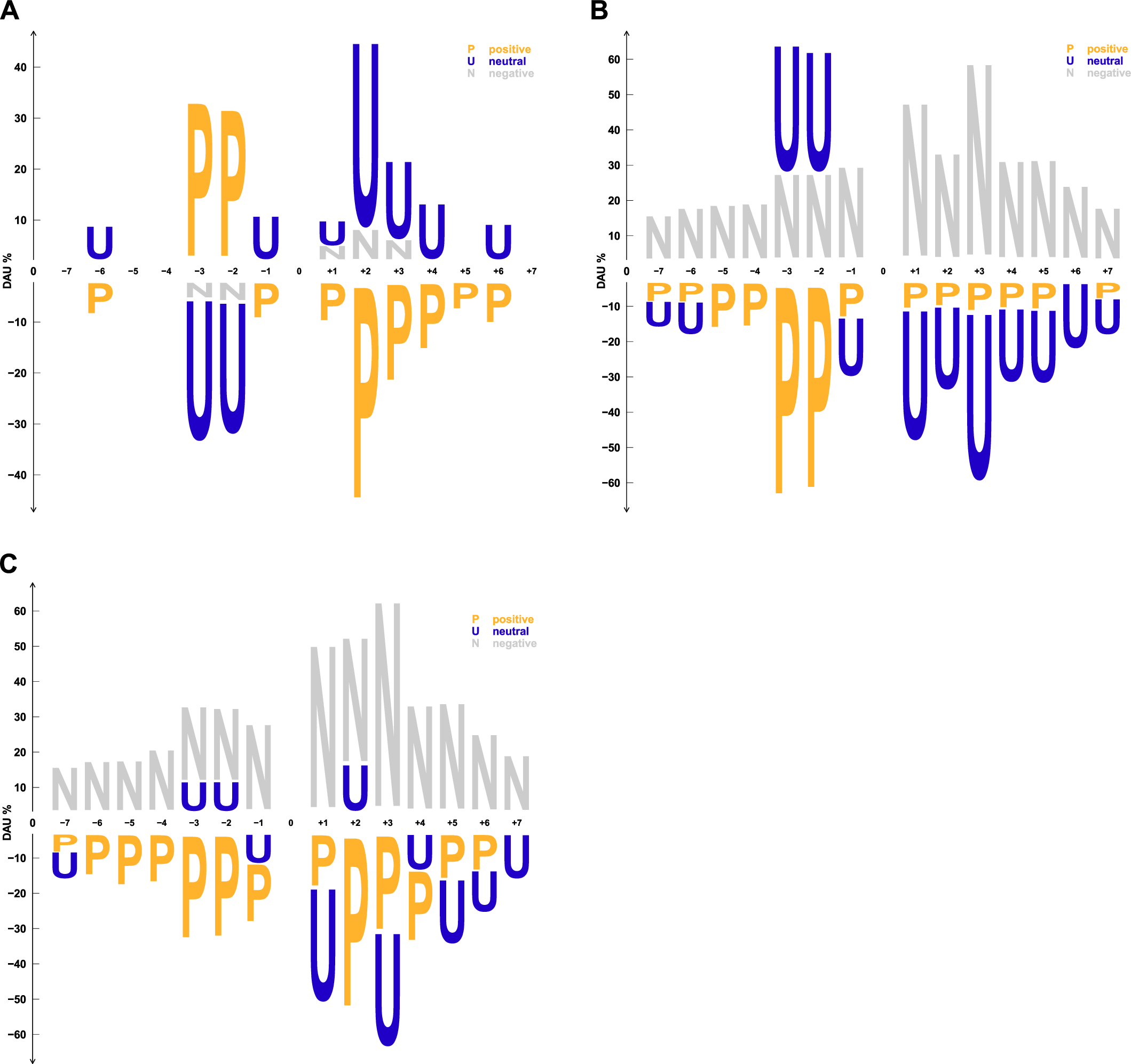

Supplement: S2 Fig — Subsequences of 15 AA residues (-7 to +7) centered on the substrate phosphorylation sites of two kinases were used as the input set and the background set, respectively. AA residues were grouped by their charge status under physiological conditions, and the differential usage of AA groups was tested by using the function testDAU to perform Fisher’s exact test with a significance level of 0.05. Positively charged AAs = {H, K, R}, neutral AAs = {A, C, F, G, I, L, M, N, P, Q, S, T, V, W, Y}, and negatively charged AAs = {D, E}. (A-C) Logos showing differential substrate AA residue group preferences of PKACA over PKCA, CK2A1 over PKACA, and CK2A1 over PKCA, respectively. (TIF) [file pone.0242030.s002.tif]

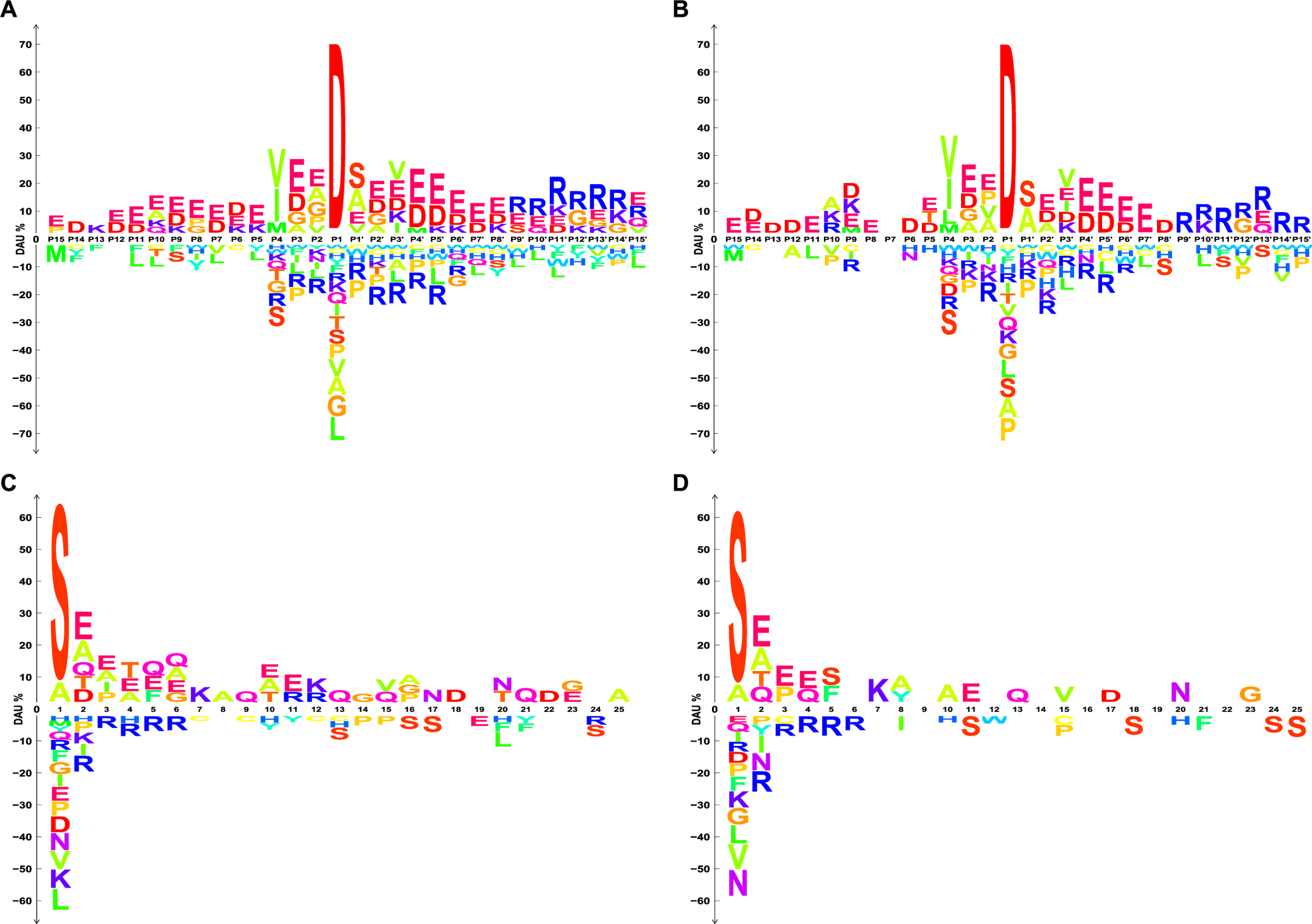

Supplement: S3 Fig — Fisher’s exact test (A and C) and Z-test (B and D) were performed to identify substrate sequence preferences of human GRB and yeast NatA, with a significance level of 0.05. For human GRB substrate specificity analyses, the same set of 416, equal-length subsequences of 30 residues centered on the cleavage sites was used as the input set, while background models for Fisher’s exact test and Z-test were built from all subsequences and from randomly sampled subsequences of 30 AA residues from the UniProt human reference proteome, respectively. For yeast NatA substrate specificity analyses, the same set of subsequences of 25 residues from the N-termini of the 285 NatA substrates was used as the input set, while background models for Fisher’s exact test and Z-test were built from all subsequences and from randomly sampled subsequences of 25 AA residues from the N-termini of the yeast proteins not including the 285 NatA substrates, respectively. The significance level was set at 0.05 for all tests. (TIF) [file pone.0242030.s003.tif]
